# Long-term results of cemented total hip arthroplasty in patients younger than 30 years and the outcome of subsequent revisions

**DOI:** 10.1186/1471-2474-14-37

**Published:** 2013-01-22

**Authors:** Marloes WJL Schmitz, Vincent JJF Busch, Jean WM Gardeniers, Jan CM Hendriks, René PH Veth, B Willem Schreurs

**Affiliations:** 1 Department of Orthopaedics, Radboud University Nijmegen Medical Centre, Internal Post 357, PO Box 9101, Nijmegen, HB 6500, The Netherlands; 2 Department of Epidemiology and Biostatistics, Radboud University Nijmegen Medical Centre, P.O. Box 9101, Nijmegen, HB 6500, The Netherlands

**Keywords:** Total hip arthroplasty, Impaction bone grafting, Young, Revision THA, Cement, Bone, Hip

## Abstract

**Background:**

The number of total hip arthroplasties in patients under 30 years is increasing over the years. Almost all of them will face at least one or more future revisions in their life. Therefore, the implant used should have a high survival rate, and needs to be easily revisable resulting in a low re-revision rate. Several studies have evaluated the outcome of total hip arthroplasties in patients under 30 years. However, only a few reported on the follow-up outcome of 10 years or more. In addition, none of these reports published data of the subsequent revisions of these implants within their original report.

**Methods:**

We studied historically prospective collected data of 48 consecutive patients (69 hips) younger than 30 years, treated with a cemented primary total hip prosthesis between 1988 and 2004. Since the last evaluation of this cohort, two patients were lost to follow-up. For all hip revisions in this cohort, again cemented implants were used, mostly in combination with bone impaction grafting. Kaplan-Meier survival curves at 10- and 15 years for the primary total hip arthroplasties and revisions were determined.

**Results:**

The mean age at time of primary surgery was 25 years (range, 16 to 29 years). Mean follow-up of the primary hips was 11.5 years (range, 7 to 23 years). During follow-up 13 revisions were performed; in 3 cases a two-stage total revision was performed for septic loosening and 9 cups were revised for aseptic loosening. There were no aseptic stem revisions. The 10 and 15-year survival rates with endpoint revision for aseptic loosening of the primary total hip were 90% (95% CI: 79 to 96) and 82% (95% CI: 65 to 92) respectively. None of our 13 subsequent revisions needed a re-revision within 10 years after re-implantation.

**Conclusions:**

Cemented total hip implants in patients under 30 years have an encouraging outcome at 10 and 15 years after surgery in these young patients. The 13 revised hips, treated with bone grafting and the third generation cement technique, were performing well with no re-revisions within ten years after surgery.

## Background

The number of total hip arthroplasties in very young patients is rising. Different solutions to treat these young patients with secondary osteoarthritis have been used over the last decade. Non-cemented, cemented, hybrids, metal-on-metal total hip arthroplasties and resurfacing prostheses all have been used in young patients, with different success rates [1-22]. Obviously, in these very young patients it is important to use total hip implants with a proven long-term outcome [23]. Additionally, we have to keep in mind that these young people will inevitably face one or more revisions in their life, due to their longer life expectancy. As a result, the implant used should have an acceptable and reliable long-term clinical outcome and it needs to be easy revisable without creating a bone stock defect. Both survival of the primary THA and subsequent revision THA are important outcome measurements in these young patients. So far, only a few reports present long-term results of total hip arthroplasties in patients under 30 years and none present the outcomes of the first revision THA’s in the same cohort (Table 1).

**Table 1:** Characteristics and survivorship of THA in patients 30 years or younger at time of operation with a mean follow-up of more than ten years in literature up to March 2011

**Study**	**No hips**	**No patients**	**Mean age in years (range)**	**Mean follow-up in years (range)**	**Survivorship/revised hips for all reasons**
**Cemented**					
Cage et al. [4]	29	17^*^	18.4 (15–21)	10.6 (8–15)	1 revision at 11 yrs
Chmel et al. [7]	66	39	19.9 (11–29)	15.1 (11–22)	70% at 15 yrs (acetabular revision)
Sochart and Porter [8]	83	55	24.9 (17–29)	20 (5–30)	89% at 10 yrs ; 65% at 25 yrs
Torchia et al. [6]	63	50	17 (11–19)	12.6 (1.6-18.6)	27 revisions^#^
Witt et al. [3]	96	54	16.7 (11–27)	11.5 (5–18)	24 (25%) at average 9.5 years follow-up
Wroblewski et al. [18]	39	28	17.9 (12–19)	12.6 (2.3 – 29.0) for non-revision	16 revisions at mean 19.1 yrs (8–34)
(current study)	69	48	24.6 (16–29)	11.3 (2–23.4)	86% at 10 yrs; 75% at 15 yrs
**Uncemented**					
Wangen et al. [16]	49	44	25 (15–30)	13 (10–16)	24 revisions of acetabular component^#^

*mixed group of patients with total hip and total knee prosthesis.

^#^at mean follow-up.

Although cemented total hip implants are not commonly used in young patients, we have always used cemented total hips in all ages, including patients younger than thirty years. Furthermore, we have one essential addition in young patients: in case of substantial acetabular bone stock deficiencies, we have reconstructed this bone stock loss using impaction bone grafting (IBG). In these young patients, secondary osteoarthritis resulting from underlying diseases is often seen in combination with associated loss of the acetabular bone stock (e.g. in developmental dysplasia of the hips and juvenile rheumatoid arthritis). This hampers an optimal insertion and fixation of the cup. We believe that IBG for the reconstruction of acetabular bone stock deficiencies in young patients is a biologically attractive approach, which restores bone stock already before the inevitable future revision [17].

In all revisions we applied the same philosophy of using IBG on the acetabular side with a cemented cup. In case of femoral bone stock loss, femoral grafting was performed as well in order to achieve a durable reconstruction [24].

The purpose of the current study is to present the clinical and radiographical results of 69 consecutive cemented total hip arthroplasties in 48 patients less than 30 years old after a mean follow up of more than 10 years, reporting the survival data with endpoint revision for any reason, aseptic loosing and radiological failure. In addition to the results of the primary total hip arthroplasties, the current state of our revisions and re-revisions within this group after the primary total hip arthroplasty will be assessed. Results will be compared to results of similar or other techniques found in the literature.

## Methods

In 2011, at a minimum follow-up of 7 years (7 to 23 years), we performed a historical prospective study on 48 consecutive patients (69 hips) younger than 30 years, who received a primary cemented total hip arthroplasty between April 1988 and May 2004. All indications except reconstructions for tumors were included. There was no selection bias as we used cemented total hips in all cases. The group consisted of 32 female (46 hips) and 16 male patients (23 hips). The mean age at surgery was 25 years (range, 16 to 29). Four patients (6 hips) died during follow-up of causes not related to their hip surgery. The mean age during surgery of these 4 patients was 24 years (range, 20 to 26). Their mean follow-up until death was 9 years (range, 1 to 18). None of them was revised before death and all of them were followed up to their death on a regular base. Therefore, we included all their data. Two patients (3 hips) were lost to follow-up since our first evaluation of this cohort, including one patient with 2 THA’s after 3.5 years of follow-up of both hips and one patient with one THA after 11 years [17]. Their data were included in the study up to their latest clinical and radiographic control. Table 2 lists the indications for the THA in all patients.

**Table 2:** Indication for total hip arthroplasty (n = 69)

**Indication**	**Number of hips**
Juvenile rheumatoid arthritis	18
Osteonecrosis of femoral head	21
*Systemic lupus erythematosus*	*7*
*Acute lymphatic leukemia*	*3*
*Crohn’s disease*	*3*
*Nepropathy, kidney transplantation*	*2*
*Hypothalamic disorder*	*1*
*Aplastic anemia*	*1*
*Wegener’s disease*	*1*
*Unknown origin*	*3*
Developmental dysplasia of the hip	7
Multiple epiphyseal dysplasia	2
Legg-Calvé-Perthes disease	6
Ankylosing spondylitis	5
Morquio’s disease	2
Ankylosis of unknown origin	2
Polycystic disease of the femoral head of unknown origin	2
Arthritis and osteomyelitits	2
Posttraumatic osteoarthritis	1
Psoriatic arthritis	1

In all cases, a posterolateral approach without a trochanteric osteotomy was used. Although we used different implants over the years, all of them were fully cemented (Table 3). All cups were made of conventional polyethylene and only cobalt-chrome femoral heads of 22 mm (9 hips), 28 mm (49 hips) and 32 mm (11 hips) were used. Up to 1990, all femoral components were inserted using a second-generation cementing technique, while from 1990 onwards the third cementing technique was used. Palacos cement was used until 1989 (Merck, Darmstadt, Germany); from 1989 on we used Surgical Simplex (Stryker-Howmedica, Newburry, UK). Antibiotics (cefazolin) were given during a maximum of 24 hours postoperatively to prevent infections.

**Table 3:** Types of implant

**Type of acetabular implant**	**Number of hips**	**Type of femoral implant**	**Number of hips**
Müller®/Allopro® cup	14	M.E. Müller® straight stem	11
Elite™ Plus LPW	26	Charnley Elite (Plus) stem™	13
Exeter/Contemporary™	29	Exeter Stem™	45

**Figure 1:**
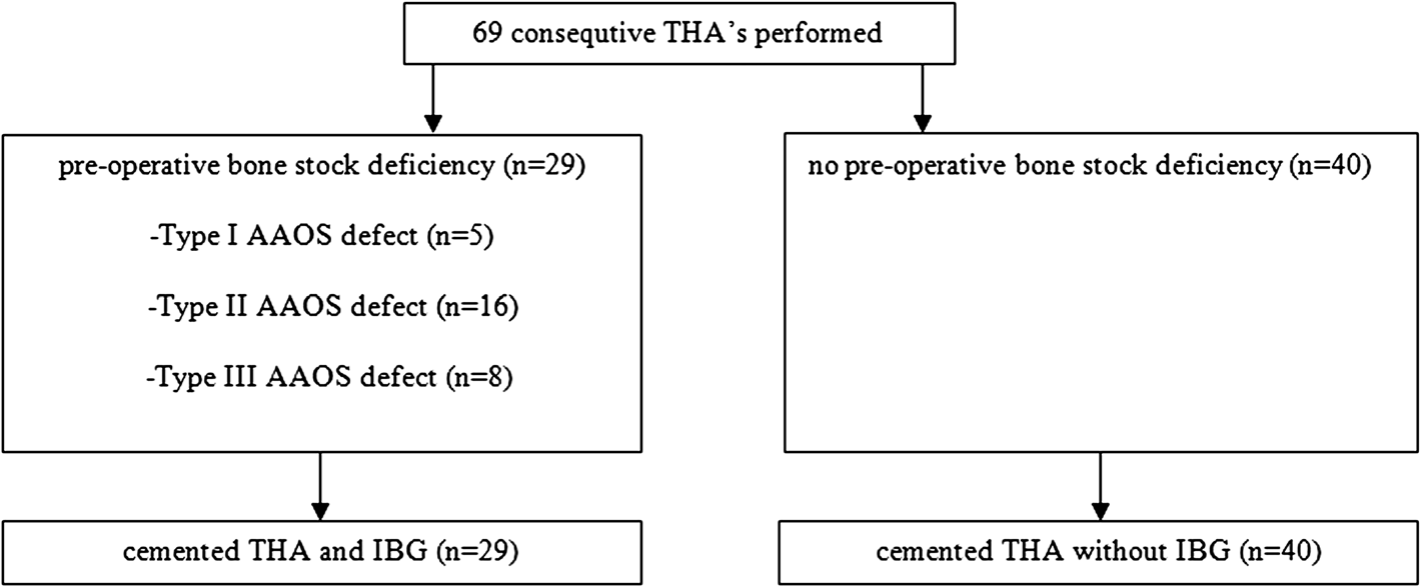
In 29 of the 69 cases, a pre-operative bone stock deficiency on the acetabular side was present, which was classified according to the AAOS classification and treated with a cemented THA and IBG.

**Figure 2:**
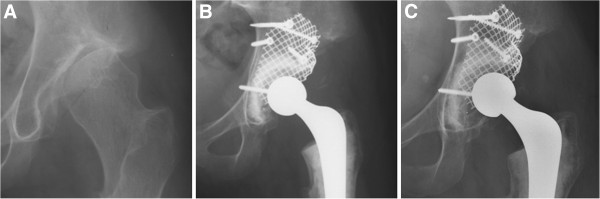
**Acetabular reconstruction in a 27-year-old woman with secondary osteoarthritis due to congenital hip dysplasia.** Preoperative (**A**), immediately postoperative (**B**), and 12 years postoperative (**C**).

In case of bone stock deficiency during the primary procedure, we used the IBG technique to reconstruct acetabular defects (29 hips) (Figure 1 and 2). This technique has been described in the literature in detail before [17,25]. Acetabular defects were classified according to the classification system of the American Academy of Orthopaedic Surgeons (AAOS) Committee on the Hip [26]. In 5 hips a Type I defect was present. A Type II defect was seen in 16 hips and a Type III defect in 8 hips. In 23 hips, femoral head autografts were used, whereas fresh-frozen nonirradiated femoral head allografts were used as a source for bone chips in 3 hips with a Type III defect. In another 3 hips, both autografts and a fresh frozen allograft femoral head were used.

Postoperatively, patients were treated with subcutaneous low-molecular-weight heparin for 6 weeks. Before 1999, oral anticoagulants were prescribed for 3 months according to our previous postoperative protocol. Indomethacin was administered for 7 days to prevent heterotopic ossification. Alternatively, one dose (7 Gy) of radiotherapy was given, when indomethacin was contraindicated. Patients without a defect or a simple minor cavitary defect were mobilized under the supervision of a physiotherapist two days after surgery. In case of extended reconstructions, 10% touch weight bearing with crutches was allowed for 6 weeks, followed by 50% touch weight bearing for another 6 weeks, before full weight bearing was allowed. In case of very extensive acetabular reconstructions, e.g. severe pelvic dislocation and high dislocation, patients initially had a six-week period of bed rest to achieve full graft incorporation before starting mobilization.

The antero-posterior pelvic and lateral hip radiographs of all hips were analysed on a consensus basis by two of the authors (MWJLS and BWS). Follow-up radiographs were complete for all hips, except for the two patients lost to follow-up. Graft incorporation was determined as the manifestation of a regular radiodensity and trabecular bone structure throughout the graft and host bone with a continuous trabecular pattern according to Conn et al. [27]. Radiolucent lines were described if they were more than 2 mm wide and were defined as stable or as progressive lines in time. The criteria of DeLee and Charnley were used to identify acetabular zones [28]. Radiographic failure was defined as radiolucent lines in all three zones and/or migration of 5 mm or more in any direction on the AP-pelvic view relative to the interteardrop line. The classification of Brooker et al. was used to describe heterotopic ossification [29]. Polyethylene wear was calculated using the method of Dorr and Wan [30]. On the femoral side, the Gruen classification was used to determine radiolucent zones [31]. Femoral prosthetic subsidence was considered if it was more than 2 mm [32] while definite loosening of the stem was defined as the appearance of a radiolucent line in all Gruen zones that did not exist on the immediate postoperative radiographs, or as a crack in the cement or fracture of the stem [33].

The Harris Hip Score and Oxford Hip Score were determined at each follow-up visit to evaluate clinical results, as were the VAS scores (on a scale of 1 to 100) for pain during rest and activity as well as the VAS score for satisfaction about the hip function (on a scale of 1 to 100).

Cemented total hip arthroplasties with acetabular IBG were used in al revision procedures. In case of femoral bone deficiency, we performed femoral IBG [34].

For this study, no ethical approval was required by the local ethical committee.

### Statistical analysis

Kaplan-Meier survival analysis were used to determine the survivorship of the primary hip implant for the endpoints revision of one or both components for 1) any reason, 2) aseptic loosening and 3) radiographic failure. In addition to analysis for the total group, hips with and without acetabular IBG were also analysed separately. The HHS and OHQS were evaluated in 3 categories: 1) after 2 to 5 years follow-up, 2) after 5 to 10 years follow-up and 3) after more than 10 years follow-up. A ‘last observation carried forward analysis’ was done to determine the mean HHS and OHQS scores for the total group of surviving hips. VAS scores regarding pain in rest, pain during activity and overall satisfaction were determined. In addition to the results of our primary total hip arthroplasties, a Kaplan-Meier survival analysis was performed for the revisions in our group. HHS and OHQS, as well as VAS-scores were also calculated for the revised total hip arthroplasties. All calculations were made with SPSS 18.0.2 and SAS for Windows 9.2.

## Results

### Clinical results

Mean duration of follow-up of all 69 primary total hips was 11.5 years (range, 7 to 23 years). During follow-up, 5 patients died (7 hips), two patients were lost to follow-up (3 hips) and 13 hips were revised for several reasons. The average pre-operative HHS score was 47.0 (range 15 to 81) (n = 30), and the average pre-operative OHQS was 43.2 (range 34 to 52) (n = 9). At review the mean HHS of the 49 surviving hips was 88.3 (n = 48) (range 55 to 100) and the mean OHQS was 17.5 (range, 12 to 34) (n = 49) after a mean follow-up of 11.6 (range 3.0 to 22.0). The mean VAS score for pain in rest and during activity on a scale from 0–100 after a mean follow-up of twelve years were 5.5 and 16.0, respectively. VAS score for overall satisfaction was 88.8.

### Revisions and complications

Three total revisions for septic loosening were performed at 4.8, 5.7 and 6.1 years after the index surgery. All were treated with a two stage treatment protocol and all components were exchanged. One patient, originally planned for a one-stage cup revision after 13 years for aseptic loosening had positive cultures at revision, suggesting septic loosening. This patient was treated during 3 months with antibiotics and despite partial revision, no relapse occurred. Nine cups had been revised because of aseptic loosening at 2.3, 3.0, 4.1, 4.9, 7.6, 8.6, 13.6, 14.3 and 23.6 years. Except for the three stems revised for septic loosening, no additional stems had failed. One highly polished stem had to be exchanged for a same size stem using the cement in cement technique during a cup revision due to damage to the taper caused during that cup revision. This case was scored as a re-operation but this stem exchange is not considered as a stem failure.

In addition to these revisions mentioned, one early re-operation after a primary THA was necessary due to a suspected infection. The patient recovered after débridement and antibiotic therapy without implant revision. Two early dislocations were seen. In one case an open reduction was needed. One partial femoral nerve deficit was seen, and despite surgical release, recovery was incomplete. One periprosthetic fracture, type Vancouver B1 occured, fifteen years after the primary surgery for which open reduction and internal fixation was necessary without exchange of the implant. Other complications and their outcomes are listed in Table 4.

**Table 4:** Complications of the 69 primary total hip arthroplasties, excluding the 13 revisions

**Complications**	**N**	**Outcome**
Early reoperation due to suspicion of deep infection	1	Fully recovered
Dislocation	2	One successful closed reduction, however one dislocation in a multi trauma patient needed surgical reduction
Neurological deficit	1	Femoral nerve exploration → incomplete recovery
Periprosthetic Fracture Vancouver B1	1	Plate osteosynthesis with cable grip plate system. No revision of the implant

### Radiographic results

Radiolucent lines around the cup were observed in 18 of the 56 surviving hips, which were progressive in 6 hips. Osteolysis was seen in different zones on the acetabular side as well as on the femoral side. The complete detailed radiographic characteristics found in the 56 non-revised total hip arthroplasties are shown in Table 5.

**Table 5:** Radiographic characteristics of 56 primary total hip arthroplasty patients that were not revised at last follow-up

**Radiographic characteristics**	
Acetabular radiolucent lines	18
* Progressive*	*6*
* Stable*	*12*
Osteolysis	9
* Zone I*	*3*
* Zone II*	*3*
* Zone III*	*2*
* Zone I + II*	*1*
Socket migration	-
Progressive tilting of the cup	-
PAO’s	13
* Brooker class II*	*9*
* Brooker class III*	*4*
Polyethelyne wear > 2 mm	3
Femoral radiolucent lines	3
* Zone 7*	*1*
* Zone 3 and 5*	*1*
* 6 out of 7 zones*	*1*
Rounding off of the calcar	8
Cortical atrophy	2
* Zone 4*	*1*
* Zone 7*	*1*
Cortical hypertrophy	5
* Zone 3*	*1*
* Zone 4*	*3*
* Zone 5*	*1*
Osteolysis of femoral component	7
* Zone 1*	*1*
* Zone 4*	*1*
* Zone 5*	*1*
* Zone 7*	*1*
* Zone 1 and 7*	*4*

Note: multiple radiographic characteristics per patient are possible.

### Survivorship analysis

**Figure 3:**
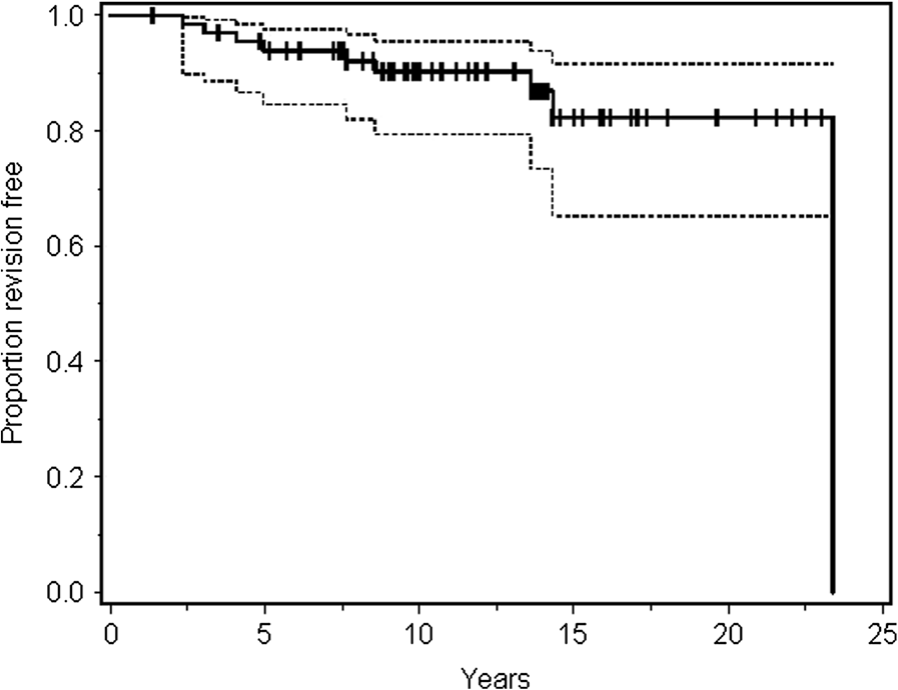
Kaplan Meier estimated survival of the total of sixty-nine hips regarding endpoint revision for aseptic loosening.

Regarding the endpoint revision for any reason of both cup and stem, the 10-year survival rate for the primary total group was 86% (95% CI: 74 to 92 ) and the 15-year survival rate was 75% (95% CI: 59 to 86) (Table 5). For endpoint revision for aseptic loosening, the 10- and 15-year survival rates for the total group were 90% (95% CI: 79 to 96) and 82% (95% CI: 65 to 92), respectively (Table 6 and Figure 3). Of the 9 revisions for aseptic loosening, 7 were primary cemented hips without IBG while there were only 2 revisions in the IBG group. The 15-year survival rate for the primary cemented hips was 80% (95% CI: 60 to 91) for endpoint aseptic loosening, whereas this was 86% (95% CI: 48 to 97) for the total hips that were combined with IBG group. This was not statistically different (p = 0.30). Survival curves for each type of implant separately did not show any significant differences.

**Table 6:** The 10-year and 15-years survivorship with the 95% confidence intervals (CI) for the three endpoints (revision for any reason, revision for aseptic loosening, radiographic failure) of the 69 hips including 29 total hip arthroplasties with impaction bone grafting (IBG) and 40 primary cemented total hip arthroplasties

**Category of patients**	**Proportion revision free for any reason**		**Proportion revisions free for aseptic loosening**		**Proportion free of radiographic failure**	
	**10-year (95% CI)**	**15-year (95% CI)**	**10-year (95% CI)**	**15-year (95% CI)**	**10-year (95% CI)**	**15-year (95% CI)**
All hips (n = 69)	86% (74 to 92)	75% (59 to 86)	90% (79 to 96)	82% (65 to 92)	90% (80 to 96)	82% (65 to 92)
Primary cemented hips (n = 40)	81% (64 to 91)	71% (50 to 84)	86% (70 to 94)	80% (60 to 91)	86% (70 to 94)	80% (60 to 91)
Acetabular IBG (n = 29)	93% (74 to 98)	83% (49 to 95)	96% (77 to 99)	86% (48 to 97)	96% (77 to 99)	86% (48 to 97)

### Outcome of the thirteen revisions

All 13 failed primary hips were revised in our institution and the reasons for revision have been explained previously. In all 3 septic loosenings, acetabular bone impaction grafting and a cemented implant was used in the second stage. Additionally, in 2 of the stem revisions femoral bone impaction grafting was used. No relapse of infection occurred. In the 9 isolated cup revisions, acetabular bone impaction was used in combination with a cemented cup. In one case the new cup was simply re-cemented. The 12 defects seen on the acetabular side were five segmental defects (AAOS type I), two cavitary defects (AAOS type II) and five combined defects (AAOS type III). All of the segmental defects were reconstructed using a wire mesh. Of the cavitary defects, one did not need any mesh while the other was reconstructed with a medial and a rim mesh. Of the combined defects, 2 received only a rim mesh and three received both a rim and a medial mesh. The mean follow-up of the 13 revisions after the surgery was seven years (range, 1 to 15 years). Remarkably, the only re-revision in this study was performed 13 years after cup revision without acetabular bone impaction grafting. One patient had a partial lesion of the femoral nerve after a cup revision, which recovered partially without any invasive treatment.

Of the twelve non re-revised cups, we collected the postoperative HHS and OHQS after a mean follow-up of 7 years (range, 0.5 to fifteen years), which were 82.7 (range 49 to 100) and 22.3 (range 12 to 52), respectively. Further analysis of these scores revealed that two patients had strikingly disappointing HHS and OHQS scores. One of the patients has a history of sclerodermia, M. Crohn, and recently received a stoma. The second patient post-operatively experienced problems of the femoral nerve which influences his activity and pain scores. This explains the differences of the HHS and OHQS scores between the primary and revision total hip arthroplasties. VAS scores of the twelve patients after revision for pain during rest and activity were 11.7 (range, 0–80) and 36.7 (range, 0 to 100), respectively. The mean VAS satisfaction score was 68.3 (0 to 100).

The Kaplan Meier survival curve of our revisions with endpoint revision for any reason showed a 100% survival at 10 years.

## Discussion

Very young patients with osteoarthritis of the hip are still a challenge for orthopaedic surgeons. Frequently, due to the underlying pathology, an acetabular bone stock deficiency in this group is seen. Often large sized cups are used in order to cover this defect, without restoring the bone stock. However, in these younger THA patients, future revisions will be inevitable. It is therefore of extreme importance to create an optimal bone stock situation in order to prepare future (re)-revisions. We have described both the technique and outcome of the reconstruction of acetabular defects with IBG in young patients up to the age of 50 before [25,35,36].

Although it is not very common in young patients, we have always used cemented total hip arthroplasties and cemented revisions in this young population. Furthermore, we used bone grafts in all cases with bone stock loss, thereby facilitating a biological reconstruction. Our 10 year Kaplan Meier estimated survival for endpoint aseptic loosening of 90% (95% CI: 79 to 96) is within the NICE criteria [37]. Our 15 year estimated survival of 82% is still acceptable in these very young patients but needs to be monitored closely over the next years.

At review of the literature in March 2011, there was only limited literature available regarding the survival and complications of a total hip arthroplasty after a mean follow-up of 10 years or more in patients under 30 years. None of the reports included the outcome of their revisions in the same report as well. It should be noted that for young patients both outcome of the primary and revision THA, are equally important. Moreover, we assume that the outcome of the revision THA can be influenced by the primary THA procedure. Our results are at least comparable to the cohorts treated with a cemented THA, mentioned in Table 1. Compared to the studies reporting on cohorts of the same size there seems to be a trend that the survival rates we obtain by using the biological IBG technique are more favourable, as can be seen in Table 1. For the non-cemented total hip arthroplasty, only Wangen et al. [16] reported the survival rates in patients less than 30 years old after a mean follow-up of more than 10 years. They reported 24 acetabular revisions in 49 hips at a mean follow-up of 13 years. McCullough et al. reported the survival rates in patients with a mean age of 24 years (range, 11 to 35) and showed a survival rate for the THA of 71% at 13 years [13]. Both these studies do not equal our satisfying long-term survival rates. Over time, other techniques like the resurfacing prosthesis and large head metal-on-metal THA, have been introduced to treat young people with osteoarthritis. Unfortunately, the resurfacing prosthesis has shown its limitations, even before a large number of long-term follow-up studies were finished [38]. A higher revision risk for younger female patients is observed, possibly related to their lower neck-head ratio, restricting the indications to younger males [39]. Furthermore, the formation of pseudotumors and elevated ion levels which have been described for large head Metal-on-Metal THA as well, cause limitations to the application of these types of arthroplasties [40].

The study of Girard et al. shows a very acceptable 10-year survival rate of 94.5% (95% CI: 80 to 98.6) for the non-cemented total hip arthroplasty with metal-on-metal bearings in patients younger than 30 years at time of surgery, but includes only 28 mm heads [22].

In our study we observed 13 revisions. Reasons for failure, in addition to the 4 septic revisions (3 complete revisions and one cup revision), are speculative and probably multifactorial. Two of our aseptic failures experienced a fall that could have caused accelerated loosening.

One cup that was part of a total hip arthroplasty revision for septic loosening performed thirteen years earlier was re-revised. All other twelve revised hips were reconstructed on the acetabular side with IBG. All of them are still in situ and none of them show radiographic loosening. No femoral revisions for aseptic loosening were performed, emphasizing the good results of the cemented femoral component in this specific young population. The 100% survival of our revisions at ten-year follow-up is a promising result for young people. The outcome of the primary prosthesis and the subsequent revision outcome are equally important to determine the best solution in young patients.

IBG was associated with attractive survival results both in primary THA and revisions. Of the 9 primary total hip arthroplasties that needed a revision for aseptic loosening, only 2 had received IBG. The results as shown in Table 5 clearly outpoint a trend that IBG shows better results compared to non-IBG. Even after 15 years of follow-up, the survival rate for endpoint revision for any reason is still 83% (95% CI: 49 to 95) and for aseptic loosening even 86% (CI: 48 to 97).

The number of hips in this study is acceptable with a regarding the longterm follow-up. In addition, the outcome of all revisions is known. In all patients, the same philosophy was used. As a referral centre we have included all indications except oncologic cases, which makes this an unique cohort. However, this study has some limitations. First, two patients were lost to follow-up. Often patients lost to follow-up are presented as censored cases. But as described by Murray, this might underestimate the revision rate [41]. Secondly, several different implants are used in our relatively small group of patients, however, all were cemented and all patients were treated in the same way according to our protocol. Survival curves for each type of implant separately showed no significant differences, and two of the components are still widely used (Exeter and Müller, Table 2) [42]. Furthermore, when reporting on surgery performed over a period of time, the use of different implants is almost inevitable. One can argue that 26 of our patients were treated for rheumatic diseases and therefore may be low-demand patients. However, most series on hip arthroplasty outcome in very young patients include a significant number of patients with rheumatic disease. Lastly, it would be interesting to have more information about the activity level of all patients in addition to the collected HHS, OHS and VAS scores, as these scores might not completely satisfactorily describe level and duration for sport activity etc. In addition, as this study is performed in a consecutive series of 69 THA’s performed in the period 1988–2004 it is not possible to retrospectively collect more information about the difference in pre- and postoperative activity level.

## Conclusions

The 10- and 15 year survival rates for endpoint aseptic loosening of cemented total hip arthroplasty in patients younger than 30 years are promising and indicate that this is a satisfying option for young people suffering of degenerative cartilage disease. IBG can be used to reconstruct the acetabular bone deficiency in primary as well as revision total hip arthroplasty. In our cohort none of the subsequent revisions was revised within 10 years. Particularly for young patients, reporting the survival of primary total hip arthroplasties and reporting the survival results of the revisions of the same cohort are equally important.

The study was performed in accordance with the ethical standards in the Helsinki Declaration of 1975, as revised in 2000. The study has been carried out in the Netherlands in accordance with the applicable rules concerning the review of research ethics committees and informed consent and does not need any further ethical approval.

## Competing interest

Each author certifies that he or she has no commercial associations (eg, consultancies, stock ownership, equity interest, patent/licensing arrangements, etc.) that might pose a conflict of interest in connection with the submitted article.

## Authors’ contributions

MWJLS participated in the design of the study, collected the data and drafted the manuscript. VJJFB collected the data and drafted the manuscript. JWMG collected in the design of the study, critically revised the drafts and drafted final version of the manuscript. JCMH participated in the design of the study and performed the statistical analysis. RPHV participated in the design of the study, critically revised the drafts and drafted final version of the manuscript. BWS participated in the design of the study, drafted the manuscript, participated in the coordination of the study. All authors read and approved the final manuscript.

## Pre-publication history

The pre-publication history for this paper can be accessed here:

http://www.biomedcentral.com/1471-2474/14/37/prepub
